# Plant genetic variation mediates an indirect ecological effect between belowground earthworms and aboveground aphids

**DOI:** 10.1186/s12898-014-0025-5

**Published:** 2014-10-21

**Authors:** Akanksha Singh, Julia Braun, Emilia Decker, Sarah Hans, Agnes Wagner, Wolfgang W Weisser, Sharon E Zytynska

**Affiliations:** 1 Terrestrial Ecology Research Group, Department for Ecology and Ecosystem Management, Centre for Food and Life Sciences Weihenstephan, Technische Universität München, Hans-Carl-von-Carlowitz-Platz 2, Freising 85354, Germany

**Keywords:** Aboveground-belowground interactions, Aphis fabae, Acyrthosiphon pisum, Genetic interactions, Plant genotype, Vicia faba

## Abstract

**Background:**

Interactions between aboveground and belowground terrestrial communities are often mediated by plants, with soil organisms interacting via the roots and aboveground organisms via the shoots and leaves. Many studies now show that plant genetics can drive changes in the structure of both above and belowground communities; however, the role of plant genetic variation in mediating aboveground-belowground interactions is still unclear. We used an earthworm-plant-aphid model system with two aphid species (*Aphis fabae* and *Acyrthosiphon pisum*) to test the effect of host-plant (*Vicia faba*) genetic variation on the indirect interaction between the belowground earthworms (*Eisenia veneta*) on the aboveground aphid populations.

**Results:**

Our data shows that host-plant variety mediated an indirect ecological effect of earthworms on generalist black bean aphids (*A. fabae*), with earthworms increasing aphid growth rate in three plant varieties but decreasing it in another variety. We found no effect of earthworms on the second aphid species, the pea aphid (*A. pisum*), and no effect of competition between the aphid species. Plant biomass was increased when earthworms were present, and decreased when *A. pisum* was feeding on the plant (mediated by plant variety). Although *A. fabae* aphids were influenced by the plants and worms, they did not, in turn, alter plant biomass.

**Conclusions:**

Previous work has shown inconsistent effects of earthworms on aphids, but we suggest these differences could be explained by plant genetic variation and variation among aphid species. This study demonstrates that the outcome of belowground-aboveground interactions can be mediated by genetic variation in the host-plant, but depends on the identity of the species involved.

## 1 Background

There is increasing recognition that aboveground-belowground interactions are important drivers of community and ecosystem processes, e.g. nutrient cycling [1]. Investigating the link between aboveground and belowground species is therefore important not only to understand the various interactions, but can also benefit the conservation of ecosystems and the services they provide [2]. Interactions between belowground and aboveground communities are often mediated by the plants that connect them [3]. This can also be described as an indirect ecological effect (IEE) when the outcome of an interaction is mediated by the presence of a third species (e.g. plant) [4],[5]. Plant-mediated indirect effects have now been shown for a variety of species interactions [6]–[9].

In an aboveground-belowground system, aboveground herbivores can positively influence soil communities by increasing soil nitrogen through returning organic matter as labile faecal material [10]; but, they can also have negative effects through impairment of net primary productivity via tissue removal [11], by reducing plant root growth and biomass [12],[13] and through the induction of secondary defense compounds [14]. Similarly, soil organisms have varying influences on aboveground communities. Insect root herbivores can induce nutrient changes within the foliage of the host plant and have been found to increase fecundity of leaf miners [15],[16] and aphids [15]. Belowground decomposers mobilize nutrients that increase plant quality and the fitness of aboveground herbivores [17],[18], and they can also upregulate defensive compounds in the plant which may negatively influence aboveground herbivores [19]. Root herbivores have also been shown to influence seed predators and natural enemy trophic levels, via plant-mediated interactions [20].

The mechanisms that drive plant-mediated interactions include effects on resource quality [8] and the induction of plant defenses [9]. The outcome of belowground-aboveground indirect interactions can be positive for the organisms involved, when both components respond similarly, or negative/neutral, when each component responds to different abiotic constraints or resource quality outweighs the effects of resource heterogeneity [2],[21]. Studies on plant-mediated indirect interactions have only rarely considered the role of plant genetic variation (but see [22],[23]); however, it is known that genetically-based traits in a plant lead to variation amongst individuals (e.g. for plant structure, nutritional value or defense chemicals) and these differences possibly play a role in species interactions. Genetic variation in plants is already known to influence the community structure of invertebrates, fungi and plants living on and around the focal plant [24]–[26]. This means that ecological communities associated with different plant genotypes vary and this can lead to changes in the interaction networks; for example, through host-associated differentiation via trophic cascades [27]. Genetic variation in the host-plant can also lead to genotype-by-environment interactions where the plant genotype mediates the effect of the indirect interaction [28]. For example, the effect of rhizobacteria in the soil on aphids feeding on the plant, and their parasitoids, is dependent on the specific genotype of the host plant and further, the genotype of the aphid [22],[23]. The study of the link between plant genetic variation and soil communities is still nascent and focuses on decomposer communities, but does show strong effects indicating potential strong linkages between these components [29].

Aphids are a good model species when studying aboveground-belowground interactions because they experience an intimate relationship with their plant hosts through feeding on the plant phloem-sap and thus are able to detect even slight changes in host quality [30]. Aphids often feed on only a few host plants but some are more polyphagous than others, which may lead them to be more susceptible to physiological changes in the plant than other more specialized aphids [31],[32]. Furthermore, aphids exhibit preference and performance differences among host-plant species and genotypes indicating that changes in host quality can affect fitness and host-choice traits [33]–[35]. Another good model species for studying belowground-aboveground interactions are earthworms which are known decomposer ecosystem engineers [36]. The regulation of plant performance by earthworms has been documented in a number of studies showing that earthworms can alter plant nitrogen content by enhancing nitrogen availability in the soil [37]–[39]. This is generally beneficial for plants but can have an indirect disadvantage, for example, when it leads to increased herbivory [40],[41]. Inconsistent effects of earthworms on aphids have been found; with positive, negative and no effects being published [40],[42]–[46]. These studies did not consider plant genetic variation within the system and were predominantly carried out using *Myzus persicae* aphids. We suggest that these interactions may be mediated by plant genotype and vary across aphid species.

**Figure 1:**
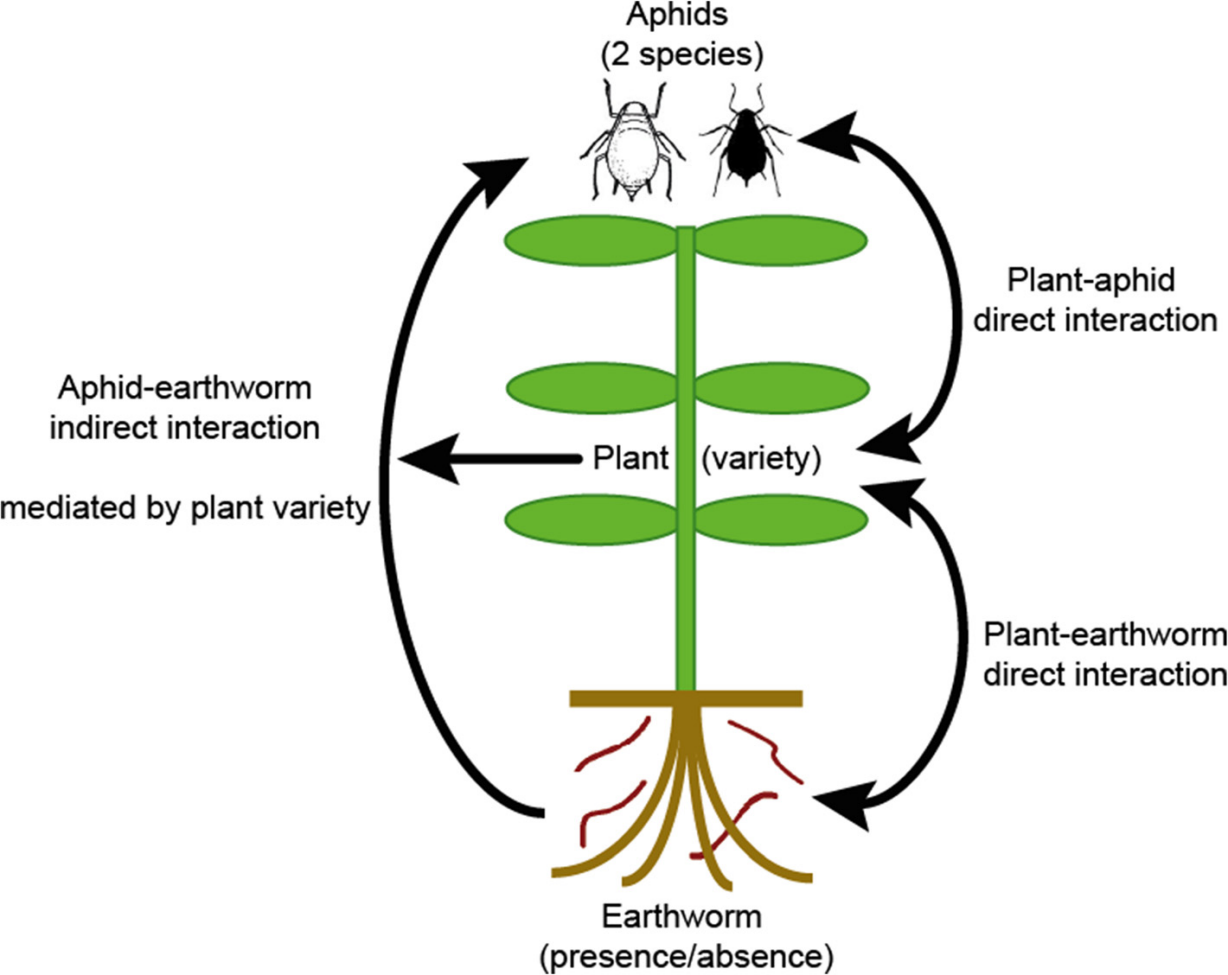
**The model system used in this study consisted of four varieties of the broad bean plant (*Vicia faba*), with two aphid species (*Aphis fabae*** **and** ***Acyrthosiphon pisum*) and earthworms (presence or absence).** Through this we were able to study direct and indirect effects of above-belowground interactions.

We conducted a greenhouse study to determine if belowground-aboveground interactions are mediated by plant variety, using a model system with composting earthworms, four broad bean plant varieties, and two species of aphid (Figure 1). The aphid species used are both common pests of bean plants, but differ in host-plant breadth (one feeding solely on legumes and the other is polyphagous, feeding on multiple plant families). We hypothesized that, earthworms would generally increase nitrogen availability in the soil, thus increasing plant biomass and also aphid density; although the magnitude of this effect would depend on the variety of the plant. In addition, we reared the two aphid species alone and together to see if competition between the aphid species would influence the effect of the earthworms on plants and aphids. The bean varieties used in our study are commercially sold broad bean varieties which are commonly grown by farmers.

## 2 Results

### 2.1 Aphid growth

**Figure 2:**
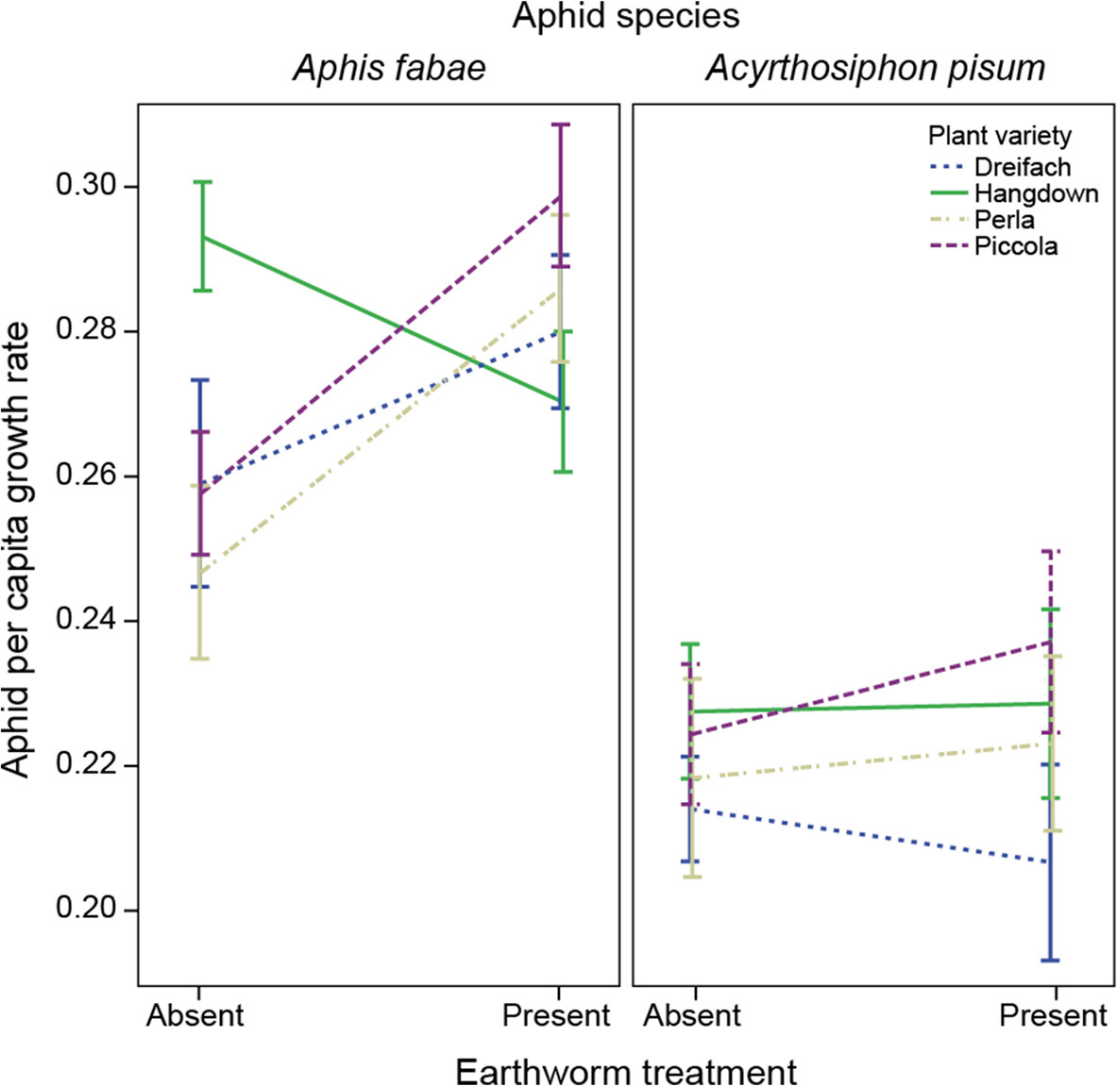
**Aphid growth rate as a function of plant variety and earthworm presence in the experiment.** Aphids were counted after 14 days and growth rate calculated separately for each species. There was a significant effect of the plant-by-earthworm interaction on the growth rate of *A. fabae*. Error bars represent ±1 SE.

Overall, the two aphid species differed in their reproductive performance (F_2,249_ = 32.42, *P* < 0.001; Table 1) with fewer *A. pisum* aphids than *A. fabae* aphids after two weeks growth (t = 7.55, *P* < 0.001). When there were only *A. pisum* we observed 206.9 ± 22.4 (mean ± SE) aphids at the end of the experiment, whereas in the pots with only *A. fabae* there were 353.5 ± 22.2 (mean ± SE) aphids, and when both aphid species (mixed) were present we observed an intermediate level with 304.4 ± 19.2 (mean ± SE) aphids*.* Further, the effect of the earthworm treatment on aphid numbers was dependent on the plant variety (worm x plant interaction: F_3,249_ = 3.42, *P* = 0.018). However, this interaction effect was not consistent across the two aphid species (Table 1; Figure 2). There was a significant effect of the worm-by-plant variety interaction on the growth rate of *A. fabae* (F_3,170_ = 4.51, *P* = 0.005) but no effect on *A. pisum* (F_3,156_ = 0.26, *P* = 0.856).

**Table 1:** Effects on aphid growth rate, for all aphids combined and each separate species

**Response variable:**	**All aphids**			***A. fabae***			***A. pisum***		
**Aphid density**	**df**	**F**	**P**	**df**	**F**	**P**	**df**	**F**	**P**
Block	3,249	11.85	<0.001	3,170	4.05	0.008	3,167	10.53	<0.001
Plant biomass	-	-	-	-	-	-	1,167	9.97	0.002
Plant variety	3,249	1.87	0.136	3,170	1.07	0.362	-	-	-
Aphid treatment	2,249	32.42	<0.001	-	-	-	-	-	-
Worm treatment	1,249	3.98	0.047	3,170	7.22	0.008	-	-	-
Plant variety x worm	3,249	3.42	0.018	3,170	4.51	0.005	-	-	-

Notes: ‘-‘shows where a term was not retained in the minimal adequate model. Models used were linear models in R. All interactions between plant, aphid, and worm treatment were tested. Aphid treatment for combined shows the difference between the species and when each species is analysed separately it shows the effect of competition from being reared together with the other aphid.

For *A. fabae* we found that for three of our four plant varieties the presence of earthworms in the system increased the growth rate of the aphids, but for one variety (Hangdown) the growth rate decreased (t = 2.08, *P* = 0.038, Figure 2). There was no effect of competition between the two aphid species, with the growth rate of neither aphid species being affected by the presence of the other (*A. fabae*: F_1,169_ = 0.24, *P* = 0.622; *A. pisum*: F_1,162_ = 0.06, *P* = 0.812).

### 2.2 Plant biomass

**Figure 3:**
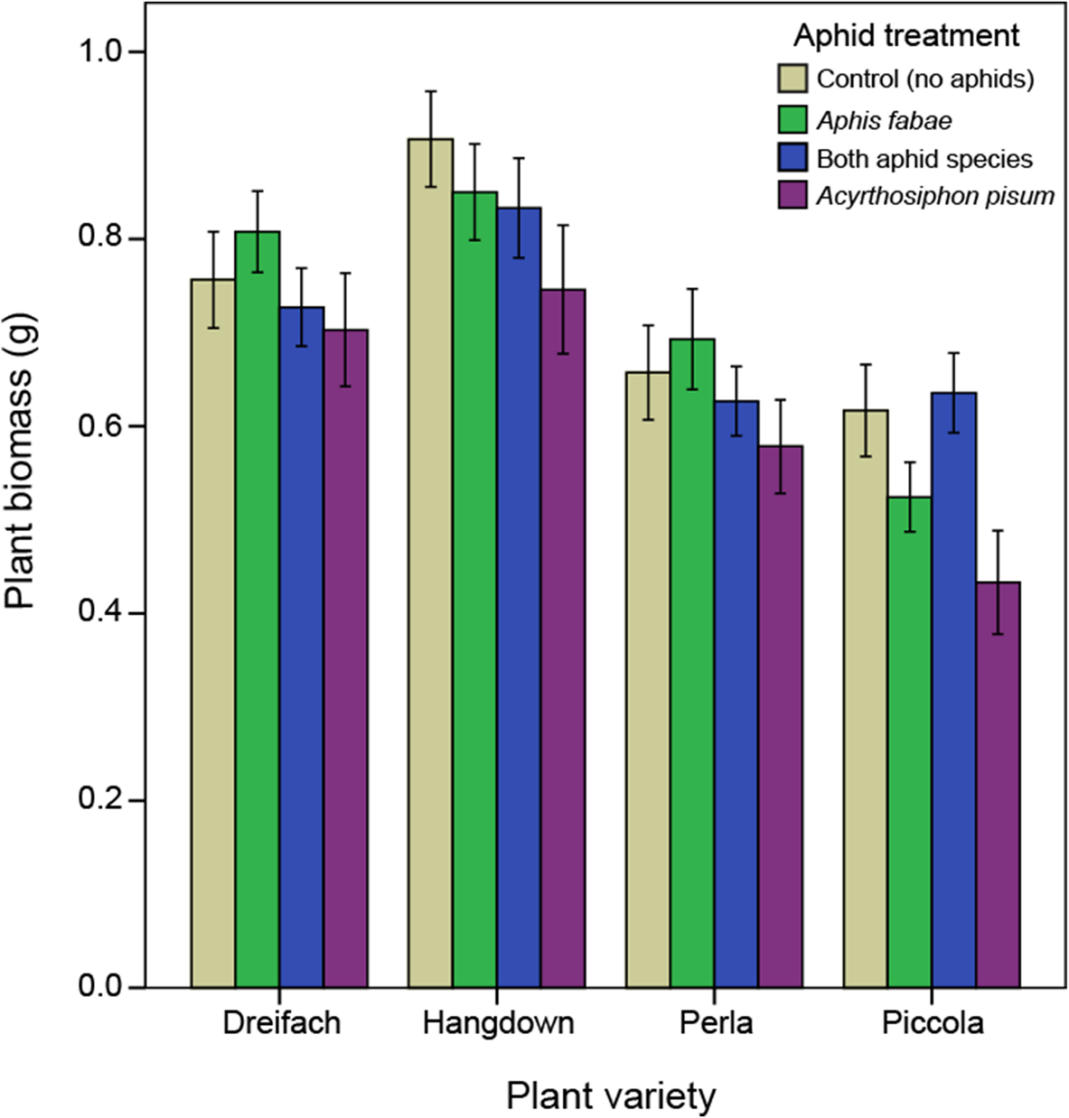
**Effect of aphid treatment on plant aboveground biomass, as a function of plant variety, after 14 days.** Plant biomass was only significantly reduced from the control (average over all varieties) when *A. pisum* was alone. Error bars represent ±1 SE.

The plant biomass varied across plant variety (F_3,339_ = 36.69, *P* < 0.001), with Hangdown producing the largest (t = 2.85, *P* = 0.005) and Piccola the smallest (t = 6.66, *P* < 0.001) plants. Earthworm presence in the soil increased plant biomass by 10.6% (across all plant varieties) (F_1,339_ = 10.07, *P* = 0.002). There was an effect of aphid treatment (F_3,339_ = 5.44, *P* = 0.001, Figure 3), with a reduced plant biomass when *A. pisum* was alone compared to the control plants with no aphids (t = 3.90, *P* < 0.001). There was no reduction in plant biomass when both aphids were present (t = 0.70, *P* = 0.485) or *A. fabae* alone (t = 0.12, *P* = 0.734). Growth rate of *A. pisum* also influenced plant biomass depending on the plant variety (F_3,163_ = 9.40, *P* < 0.001); Hangdown experienced reduced biomass with increasing aphid growth rate (t = 2.35, *P* = 0.021) whereas the other plant varieties experienced no such effect.

### 2.3 Plant Carbon/Nitrogen ratio

**Figure 4:**
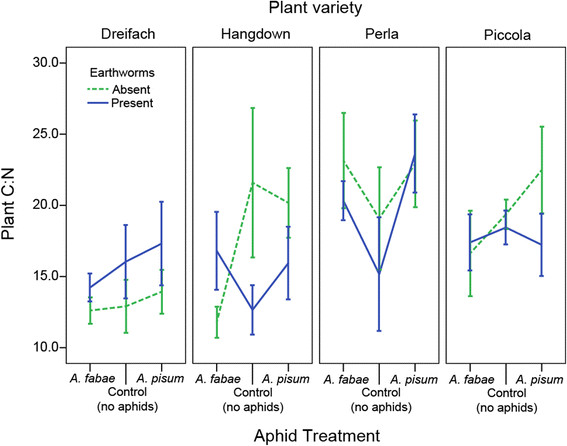
**The effect of plant variety, worm presence/absence and aphid treatment on plant C:N ratio.** There was a significant 3-way interaction between all factors on plant C:N. Error bars represent ±1 SE.

Plant C:N did not influence aphid density (all aphids: F_1,136_ = 0.007, P = 0.935) but it was itself influenced by a three-way interaction between plant variety, earthworm treatment and aphid treatment (F_9,163_ = 2.64, P = 0.007; Figure 4). This means there was no overall negative or positive effect on plant C:N of the earthworms, with the outcome dependent on the combination of plant variety and aphid treatment (Figure 4). This interaction term was partly driven by differences in the Hangdown variety across the worm and aphid treatments (t = 2.24, P = 0.026), where the C:N was lower (increased nitrogen) when earthworms were present in control and *A. pisum* treatments, but the opposite was true for the *A. fabae* treatment (Figure 4). In addition, there was a higher C:N (reduced nitrogen) in Perla when aphids were present than when aphids were absent. Overall the plant varieties, Dreifach and Hangdown had lower C:N than Perla and Piccola.

## 3 Discussion

In this paper we have demonstrated an effect of belowground earthworms on aboveground aphid growth that was mediated by plant genetic variation and differed among aphid species. On three of the four plant varieties tested, the presence of earthworms increased the number of *A. fabae* aphids, whereas on one (Hangdown) there were more aphids when the earthworms were absent. These effects were only found for *A. fabae* and not for *A. pisum* aphids. *Aphis fabae* aphids had no reciprocal effect on the plant biomass whereas the presence of *A. pisum* aphids was found to reduce plant biomass; this was most apparent in the variety Hangdown, which in addition to the effect of *A. pisum* presence also showed a decrease in plant biomass with increasing aphid growth rate. Plant C:N had no influence on aphid growth rate, but it was itself influenced by the combination of all treatments in the experiment, showing that interactions between the above- and belowground communities can alter plant chemistry.

The interaction outcome between *A. fabae* and earthworms on the plant variety Hangdown differed compared to the other plant varieties for aphid number and also for plant C:N. The control plants and those with *A. pisum* had lower C:N (increased nitrogen) in the presence of worms, which is expected since earthworms can enhance nitrogen availability in the soil [37]–[39]. When *A. fabae* aphids were on the Hangdown plants with earthworms, the C:N was increased (reduced nitrogen) and this corresponded to a reduction in aphid growth rate when earthworms were present; however, as there was no significant direct effect of plant C:N on *A. fabae* aphid growth rate other genetically-based traits must also be involved, e.g. plant-defense chemicals [9]. This work suggests that earthworm mediated changes in plant nutrients are to some extent involved in these interactions, but it is not a simple effect of changing resource availability. Since this interaction effect was only detected for the polyphagous *A. fabae* aphids, this may support the findings in other herbivores that generalists are more susceptible to changes in the plant than specialists [31],[32].

In our study, plant biomass increased in the presence of earthworms; this has also been found in previous studies, where earthworm presence increased the nitrogen content of plant roots and shoots leading to increased overall plant biomass [44],[45],[47]. The effect of earthworms on the plant chemistry in our study was found to be dependent on the plant variety and aphid treatment, such that the level of nitrogen in the plant was not consistently increased in the presence of earthworms. We also found that an increasing growth rate of *A. pisum* aphids reduced plant biomass in the Hangdown variety with no such influence for the other varieties. This was not driven by plant C:N since the ratios were similar between *A. pisum* and no aphid controls in this plant variety. *Aphis fabae* did not affect plant biomass at the densities reached in our experiment. It is expected that future aphid growth would have resulted in further detrimental effects on the plant, especially as in our experiment the aphids were unable to disperse from the experimental environment [48],[49].

In our experiments we used two different aphid species and we found that there was no effect of competition between the two species on the growth rate of aphids on the plants. It is thought that competition between closely related phytophagous species would be higher than between unrelated species due to similar resource usage [50]. Predominantly, we observed *A. fabae* on the plant stem and *A. pisum* on the leaves indicating that spatial separation may reduce competition between these aphid species. The lack of competition effects also shows that there was little resource limitation during the experiment possibly due to the use of nutrient-rich potting substrate. Our study used a legume plant grown in potting substrate that is high in nutrients and it is possible that in less nutrient-rich soils we would detect a stronger effect of the earthworms on the plants and aphids [43]. Nevertheless, earthworms did mobilize additional resources that resulted in better plant and aphid growth. Furthermore, *V. fabae* is a rhizobial species and studies have shown the nature of this legume-rhizobium mutualism to depend on factors such as abiotic nitrogen and genotype of interacting partners [51],[52]. Herbivores, earthworms and rhizobia can all influence ecological interactions involving their host plant and indirectly effect the performance of one another, due to their influence on plant resources [53]. Similarly, in our system, rhizobial associations may have differed amongst *V. fabae* varieties, which further resulted in the varied effect of earthworms on *A. fabae* on different varieties.

Previous work has found that different species of earthworms and plants have varying effects on aphid populations. Research on the combined effect of Collembola and earthworms on the development of aphids, with plants (*Poa annua* and *Trifolium repens*) grown in nitrogen-limited soil, found the outcome to vary across time periods [43]; one period in their experiment showed a 70% increase in aphids but in others there was no effect. Additionally, earthworms were found to increase aphid growth rate on *Cardamine hirsuta* in a study investigating the effect of earthworms in soils with contrasting nitrogen content on plant-aphid-parasitoid interactions [44]. However, the same lead author [45] found varying effects across different plant species when investigating the combined effects of earthworms and litter distribution of plants of different functional groups; here, earthworms reduced the number of aphids on *Plantago lanceolata* (forb) but had no effect on *Lolium perenne* (grass) or *T. repens* (legume). It was assumed that the reduced growth rate of aphids here could be the result of earthworm enhancement of defense-related secondary compounds via increased nitrogen availability to the plant [45]. These studies were all conducted with the aphid *Myzus persicae*, involved endogeic soil-feeding earthworm species (*Aporrectodea caliginosa* and *Octolasion tyrtaeum*) and were conducted on only one plant variety in each species*.* In studies on other species of aphid, no effect of *A. caliginosa* earthworms on *Sitobion avenae* aphids [40], and a decline in *Rhopalosiphum padi* under drought conditions [42], were detected. Thus, the mechanism driving any indirect effect between earthworms and aphids is far from simple and will likely depend on many other interacting abiotic (e.g. water availability; [42]) and biotic factors (e.g. presence of other soil organisms; [46]). It can also be influenced by differences amongst plant varieties and potentially mediated by plant chemistry as we have shown here.

## 4 Conclusions

We found that plant genetic variation can mediate interactions between aboveground and belowground communities. It is accepted that plants mediate aboveground-belowground interactions; however, our study emphasizes the significant role plant genotypes could play in regulating these interactions. More so, these effects are complex and species dependent. Our study showed how plant variety mediated the earthworm effect on only one of the aphid species *A. fabae,* whereas, it was the other aphid species, *A. pisum*, that reduced plant biomass. Our work adds the knowledge of how aboveground-belowground interactions are an important driver of species interactions and ecosystem processes.

## 5 Methods

### 5.1 Study system

Our system consisted of composting earthworms (*Eisenia veneta* (Rosa) formally *Dendrobaena veneta*; Lumbricidae), broad bean plants (*Vicia faba* L., Fabaceae) and two species of aphid, the legume specialist pea aphid (*Acyrthosiphon pisum* (Harris), Homoptera: Aphididae, clone FS_PA1, collected in Freising, Germany) and the polyphagous black bean aphid (*Aphis fabae* Scop. Homoptera: Aphididae, clone JAF1 originally collected in Jena, Germany). The compost earthworm *E. veneta* prefers warm and moist environments, and can rapidly consume a wide variety of compost material. The aphid species used both readily feed on the host plant (a common agricultural plant) and other legumes, although *A. fabae* is a more generalist feeder than *A. pisum*.

The earthworms were purchased from Wurmwelten, Germany (www.wurmwelten.de) and maintained in plastic boxes (7.5 cm × 15 cm) with air holes. Prior to the experimental setup, we cleared the gut contents of the worms to avoid contamination from packing soil by washing the earthworms with tap water and placing them in clean plastic boxes containing only moistened tissue for 24–48 hours at room temperature. Then the worms were sorted by size (small, medium and large) and placed into new boxes containing the experimental soil (Floragard product Floradur Topfsubstrat, pH 5.6, salinity 1.2 g/l). We used similar, ‘medium’ sized earthworms (0.2-0.4 g per worm) in the experiment.

The four plant varieties in the experiment (dreifach Weiße, Piccola, Hangdown and Perla) were purchased from Garten Schlueter, Germany (www.garten-schlueter.de). The seeds were germinated in experimental soil in pots (11 cm diameter), one seed per pot, and grown for three weeks in a greenhouse at 23/18°C (day/night) 16:8 hours (light:dark) watering daily with tap water. Experimental plants were selected by similar height and number of leaves (within variety) and kept in the same pots as the seeds were germinated in.

The experimental aphids were reared on *Vicia faba* variety *‘The Sutton*’ from Nickerson Zwaan, UK (www.hazera.com) in a climate chamber at 21°C 16:8 hours (light:dark) prior to use in the experiment. The aphids used have been maintained as clonal lines at Dürnast Experimental Station since 2011.

### 5.2 Experimental design

We used a fully factorial randomized block experimental design with two earthworm treatments (presence and absence), four plant varieties (*Dreifach Weiße*, *Piccola*, *Hangdown* and *Perla*) and four aphid treatments – two single aphid treatments (*A. pisum* or *A. fabae* alone), a paired treatment (*A. pisum* + *A. fabae* together) and a no-aphid control. This produced 32 treatments and we made 12 repeats per treatment (384 pots) over four treatment blocks, each containing three repeats. Within a block the treatments were fully randomized. Each experimental block was separated by time and all were conducted between October 2012 and January 2013 in a greenhouse with 18°C 16:8 hours (light:dark) at Dürnast Experimental Station, Technische Universität München, Freising, Germany.

### 5.3 Experimental setup

For the earthworm present treatment we added seven earthworms into the soil (placed into a small hole and covered with soil) and for the aphid present treatments, we added six 4^th^ instar or adult aphids to the bottom leaf of a plant using a fine paintbrush. To maintain the same density of aphids we added six of the same species for the single aphid treatments, or three of each species for the paired aphid treatments (substitutive design). Each plant was then covered with an air-permeable transparent plastic bag (18 cm × 30 cm; UNIPACK, Hamburg, Germany) secured by a rubber band around the pot, to stop aphid movement between plants. The pot bases were also covered using a fine mesh material and secured with a rubber band to stop earthworm movement between pots, whilst allowing for watering at the base of the plant. The pots were placed into trays (12 per tray) and watered every two days by flooding the tray; each tray had drainage holes to ensure the soil was not waterlogged. Plant height (cm) was measured before the worms were added, from the top of the seed (below the soil surface) to the terminal bud. After 14 days, the number of aphids on each plant was counted using a tally counter - we started counting (separately for each species) from the bottom of the plant, and moved upwards up to the top. The height of the plant (cm) was again recorded (top of seed, under soil surface, to terminal bud) and the plant shoot was harvested and dried at 60°C for five days, in a paper bag, after which the dry biomass was measured. The number of remaining earthworms was also counted by breaking open the root structure of the plant to recover the earthworms; they were mainly found in the dense root section of the pots.

To determine if any effect of the worm treatment on the aphids could be explained by carbon (C) and nitrogen (N) changes in the plant we analysed a subset of the samples for C and N levels. After all biomass was recorded, 198 plants were analysed with 6–8 repeats from each treatment. A 5 cm section of the dried stem was collected and ground to a fine powder using a mixer mill (MM 300; Retsch). To aid grinding, the stem tissue was manually cut into pieces using a scalpel blade (cleaned with 70% ethanol between samples) and the stem tissue was frozen at −20°C for one hour. A 2 mg sample of the ground powder from each plant sample was weighed in a tin container and then loaded into a CNH analyzer (EuroEA3000 element analyser purchased from HEKAtech GmbH).

### 5.4 Data analysis

We removed 27 pots from the analyses due to no worms remaining in the worm present pots and for a couple of pots where there was contamination of aphids across aphid treatments. An average of five earthworms was recovered at the end from each pot, with no effect of the manipulated variables on the number of earthworms recovered. This means our sample size was 350 with 8–12 replicates remaining per treatment. We calculated the aphid per capita growth rate by calculating the difference between the natural log of the number of aphids at the end of the experiment with the natural log of the number of aphids at the start, and then dividing by the number of days the aphids were on the plant. We used this method because we started with either three or six aphids, per plant genotype, depending on the aphid treatment (single or paired) and in block one the aphids were grown for 15 days whereas they were grown for 14 days in all other blocks.

The data were analysed using linear models in R v2.15.2 using R-studio v 0.97.314. Our dependent variables were aphid growth rate (one model each for all aphids, and then each species separately) and plant biomass. The independent fixed effects were earthworm presence/absence, plant variety and aphid treatment (*A. fabae*, *A. pisum*, both or none). We also added block to the model and plant biomass as a covariate for the aphid models. For the plant biomass, we also ran a model with *A. pisum* aphid growth rate, plant variety and earthworm treatment due to the results of the previous model. For the subset of data with plant CN ratios, we added these to the above models and ran another model with CN ratio as the dependent and earthworm, plant and aphid treatments as fixed effects with block (factor) and plant biomass (covariate). The minimal adequate models are presented in the results. Briefly, we first fit a full model will all main and interaction effects, and then we simplify the model by removing the non-significant terms (starting with the highest interaction term), testing to see if model fit has significantly changed. If model fit was significantly changed then the term was re-added into the model. If an interaction term was significant the corresponding main effects remained in the model, whether or not they were significant. Treatment levels were compared in R using post-hoc contrasts.

### 5.5 Availability of supporting data

The data set supporting the results of this article is available in the Labarchives repository DOI:10.6070/H42N507N. https://mynotebook.labarchives.com/share/Sharon%2520Zytynska/MjAuOHw1ODUwNy8xNi9UcmVlTm9kZS8yNjY3ODg4ODQxfDUyLjg=.

## Abbreviations

IEE: Indirect ecological effect

C:N: Ratio of carbon to nitrogen

## Competing interests

The author declares that they have no competing interests.

## Authors’ contributions

SZ designed the experiment, with AS, SZ, JB, ED, SH, AW collecting the data. Analysis and interpretation was done by AS, SZ and WW. AS, JB, ED, SH, AW all contributed to the first draft, completed by AS and commented on by SZ and WW. All authors read and approved the final manuscript.
